# Mechanical Properties and Tribological Behavior of MoS_2_-Enhanced Cellulose-Based Biocomposites for Food Packaging

**DOI:** 10.3390/polym13111838

**Published:** 2021-06-01

**Authors:** Shih-Chen Shi, Pramod Kumar Mandal, Tao-Hsing Chen

**Affiliations:** 1 Department of Mechanical Engineering, National Cheng Kung University (NCKU), No. 1 University Road, Tainan 70101, Taiwan; n16047151@mail.ncku.edu.tw; 2 Department of Mechanical Engineering, National Kaohsiung University of Science and Technology, Kaohsiung 80778, Taiwan

**Keywords:** cellulose, MoS_2_, mechanical properties, water vapor permeability, tribology

## Abstract

Synthetic polymers are the most commonly used polymers in daily life. Therefore, it is necessary to develop environmentally friendly polymers. Hydroxypropyl methylcellulose (HPMC) is a potential candidate for a biopolymer, owing to its unique properties. However, HPMC biopolymers have some disadvantages compared to synthetic polymers. In this study, the mechanical properties and tribological performance of MoS_2_ additive-enhanced cellulose matrix biocomposites were investigated in order to improve the properties of HPMC. MoS_2_ was incorporated into the HPMC matrix as a strengthening additive. The mechanical properties, bonding, and water vapor permeability of the composites were analyzed. The mechanical and vapor barrier properties of the HPMC films were significantly enhanced. The ultimate tensile strength and Young’s modulus of the composite films increased with the addition of up to 1 wt% MoS_2_. The water vapor permeability of HPMC films reduced when additives were incorporated. The wear test proves that the MoS_2_ additives can improve the tribological performance of the HPMC composite while reducing the friction coefficient. The main reason for enhanced tribological performance is the improvement in load capacity of the composite coating by the MoS_2_ additive. This MoS_2_/HPMC biocomposite can be used in food packaging.

## 1. Introduction

When food is exposed directly to air, it is not contaminated by dust easily but becomes more likely to breed bacteria and other microorganisms, which may cause diseases. Food packaging can block direct contact with air, moisture, and light and can prevent contamination during transportation and sale, thereby extending shelf life and making the food safer and more hygienic. Since the development of food packaging in response to urban development and the needs of people’s lives, it has also become necessary to consider the convenience and efficiency of food management in a large number of transport, handling, storage, sales, and eating cases. The advantages of traditional plastic packaging are its low price, low density, toughness, ease of production, and low water vapor permeability. However, the development of new food packaging materials is necessary due to ecological demands. Moreover, a food packaging material must ensure freshness, safety, and environmental protection. Some studies have employed nanotechnology and decomposable materials for food packaging [1,2]. The basic need of future food packaging materials is decomposable materials, bio-friendly materials, low light penetration, excellent gas/moisture barrier properties, and appropriate mechanical properties. At present, the common materials that meet the above requirements are cellulose [3,4,5] and chitosan [6,7,8].

Chemically modified polymers have been extensively investigated for the development of new biomaterials with excellent physicochemical properties. Cellulose is the most abundant polysaccharide found in nature. It is regular and linear and has a rigid structure because of its configuration and intramolecular hydrogen bonds. The intermolecular hydrogen bonds between the hydroxyl groups result in aggregates or crystalline forms of cellulose. This association between the molecules leads to the formation of highly ordered crystalline regions, which makes cellulose only slightly soluble in pure water [9].

Synthetic polymers are the most used polymers in daily life. However, they are non-biodegradable, difficult to recycle, and based on non-renewable materials. Therefore, the development of environmentally friendly and biodegradable polymers of natural origin that can replace synthetic polymers is necessary for environmental and resource conservation. Hydroxypropyl methylcellulose (HPMC) is an odorless and tasteless edible plant derivative with properties such as transparency, oil resistance, and the ability to form a gel upon heating. Considerable work has been conducted on the characteristics of HPMC, which possesses unique tribological properties [10], self-healing behavior [11], and features that are amenable to enhancement [12]. Furthermore, HPMC can be easily detected using standard analytical tools, which are suitable for industrial applications. However, HPMC biopolymers have some disadvantages compared to synthetic polymers such as poor tensile strength, elongation factor, and water vapor barrier performance. As a result of these disadvantages, they cannot be used in practical applications. Consequently, several studies have used additives to improve the properties of HPMC [13,14]. This study investigates the effects of molybdenum disulfide (MoS_2_) additives on mechanical properties and tribological behavior.

MoS_2_ has a low friction coefficient. When added to polymers, it forms composites with improved strength and reduced friction. Previous studies have demonstrated the use of MoS_2_ additives in applications, such as lubrication, anti-wear, anticorrosion [15], and biocompatibility [16].

The addition of appropriate quantities of MoS_2_ can enhance the tribological performance of HPMC, and the mechanism responsible for this is well-understood; MoS_2_ and HPMC will peel off from the coating surface to form a transfer layer [17]. However, the effects of additives on the mechanical properties of composite films and the relationship between these mechanical properties and tribological performance are unknown.

HPMC has potential for applications in food packaging [18,19], pediatric usage [20], and drug release control techniques [21]. Thus, analyzing the mechanical and tribological properties of HPMC films is significant. Therefore, we studied the mechanical properties of HPMC polymer films with different molecular masses, the augmentative effects of MoS_2_ addition, and their mechanisms. In addition, the factors that influence the water vapor permeability (WVP) of the composite films are discussed. The findings of this study can serve as a useful reference for the diversification of HPMC film applications. 

## 2. Materials and Methods

### 2.1. Materials

Hydroxypropyl methylcellulose (HPMC, Pharmacoat 645, 606, and 615, Shin-Etsu, Tokyo, Japan) of three grades based on viscosity and molecular weight as shown in Table 1 was used. MoS_2_ particles with an average size of 2 μm were obtained from Sigma Aldrich (St. Louis, MO, USA). The material properties of HPMC and MoS_2_ are listed in Table 2.

### 2.2. Film Preparation

The preparation of MoS_2_/HPMC composite films followed a four-step process. Initially, water and ethanol were mixed in a ratio of 1:4 in a clean flask and then heated to 60 °C. Then, 3 g of all grades of HPMC powder was poured into the solvent of 100 g and magnetically stirred at 500 rotations per minute for 12 h. Then, MoS_2_ additives (listed in Table 3) were added to HPMC solution. After preparation, the solution was stilled for 3 h for degassing and in order to prevent the formation of microbubbles within the films. A 30 g mixture MoS_2_/HPMC solution was then poured into a petri dish for film preparation and placed inside a controlled-environment chamber at 40 ± 10% relative humidity (RH) and at 30 ± 10 °C to dry the film. After 6 h, the petri dish was removed from the chamber, placed at a temperature of 25 °C with a relative humidity of 60%, and dried for 24 h. After drying, the films were removed from the Petri dish and stored under 30% RH at 25 °C. Both film thickness and surface roughness (Ra) were measured using a 3D laser scanning microscope (VK9700, Keyence, Osaka, Japan), controlled at 200 ± 10 µm and 2 ± 0.5 µm. A scanning electron microscope (SEM, AURIGA, Carl Zeiss AG, Jena, Germany) was used to observe the surface morphology of the coating.

### 2.3. Determination of Mechanical Properties

The cast films were removed and cut into a bone shape using an ASTM D-638-V “dog bone” punch. Films with cracks, nicked sides, or bubbles were discarded. The mechanical properties of the cast films were determined using a micro/nano tensile testing machine (DDS32, Kammrath & Weiss GmbH, 44,141 Dortmund, Germany) according to the ASTM D882 standard. 

### 2.4. Material Properties Analysis of Composite

The crystallization parameters were collected by a Bruker D2 Phaser diffractometer, using CuKα (Kα1, 0.15406 nm) at an operating voltage and current of 30 kV and 10 mA, respectively. The blank scan was carried out using a clean zero background plate with no applied sample. The blank scan was subtracted from the sample scan during the analysis. The intermolecular force was determined using attenuated total reflection (ATR) Fourier transform infrared (FTIR) spectroscopy (Thermo Nicolet NEXUS 470, Golden Valley, MN, USA, GMI). The WVP of films was measured according to the ASTM 1290-93 standard [28]. A unique glass cup with a diameter of 6.5 cm and a depth of 3.5 cm, with a small platform at the top to seal the films, was used to assess the WVP of the prepared films. The films were cut into discs with a diameter slightly larger than that of the cup. Distilled water (10 mL) was dispensed into the cup, which was then covered with the film and sealed with a ring. The cup was weighed and placed inside a controlled environment chamber at 30 °C and ±40 RH. The cups were weighed every 8 h to obtain at least four data points. The WVP of the film was calculated using the following equation:

WVP = (WVTR·y)/(p_1_ − p_2_), (1)

where y is the mean thickness of the film, and p_1_ and p_2_ are the partial pressures of water vapor on the lower and upper side of the film, respectively.

Water vapor transmission rate (WVTR) was in turn obtained as follows:

WVTR = weight loss per unit time/film area.(2)

### 2.5. Tribology Performance of MoS_2_/HPMC Composite Film

The tribological performance was evaluated using a ball-on-disk tribometer (POD–FM406–10NT, Fu Li Fong Precision Machine, Kaohsiung, Taiwan) under a load of 2 N and disk speed of 0.03 m/s. A chrome steel ball (52,100 steel) with a diameter of 6.31 mm was employed as the upper ball, and the composite films were employed as the lower disk test piece. The wear test was performed in an environment at 25 °C and a RH of 70%. The friction coefficient of the coating was monitored and recorded in real time, and the wear volume was measured using a 3D laser scanning microscope.

### 2.6. Third-Body Theory

The third-body theory describes the interaction of the counterpart of the materials involved in the abrasion dry friction conditions [29,30]. The velocity accommodation mechanism refers to the location and motion state of the wear as sites (S) and modes (M). S1 and S5 are called the first-bodies (representing two counter-wear parts); S3 refers to the natural wear debris generated by abrasion or lubricant additive; and S2 and S4 are the interface layers between S1, S3, and S5–S3. Mode represents the mode of velocity accommodation. M1–M4 represent elastic deformation, normal breaking/rupture, hearing/sliding, and rolling mechanisms, respectively [31].

## 3. Results and Discussion

### 3.1. Surface Morphology and XRD Analysis of Composite Film

**Figure 1:**
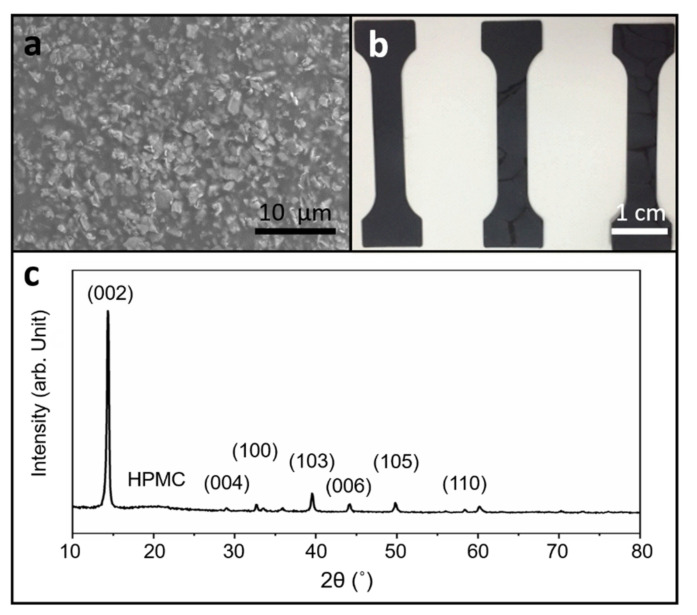
(**a**) SEM image of composite film; (**b**) samples for micro-tensile test; and (**c**) XRD analysis results of MoS_2_/HPMC composite.

The SEM image showing the surface pattern of the MoS_2_ 1 wt%/HPMC composite is presented in Figure 1a. The image shows that the additive and HPMC matrix are in good condition and that there is no obvious particle agglomeration. Figure 1b shows the micro/nano tensile testing sample made of the MoS_2_ 1 wt%/HPMC composite. The composite material is black in color, which means that the addition of MoS_2_ turns the originally transparent HPMC into black and can absorb a large amount of light, achieving the purpose of blocking light. The tribological behavior of MoS_2_ is immensely sensitive to environmental conditions. In extremely humid environments, sulfides are prone to combination with the moisture in air and form oxides, resulting in a decrease in tribological performance [32]. Meanwhile, the structure and morphology of MoS_2_ affect its abrasive performance [33]. Therefore, it is crucial to understand the structure of MoS_2_ additives in composite materials following the preparation process. The above samples were analyzed via X-ray diffraction (XRD) analysis, and the results are shown in Figure 1c. XRD patterns of the MoS_2_/HPMC composite are shown in Figure 1. The MoS_2_/HPMC composite clearly displays diffraction peaks of MoS_2_, which can be indexed to the (002), (004), (100), (103), (006), (105), and (110) planes of 2H-MoS_2_ (JCPDS card No. 37-1492). This result indicates that the preparation process does not damage the 2H-MoS_2_ additives. Meanwhile, the highly intense (002) peak indicates that the layered structure of MoS_2_ is parallel to the composite surface, which helps to discern its tribological properties.

### 3.2. Mechanical Properties of Composite Films

**Figure 2:**
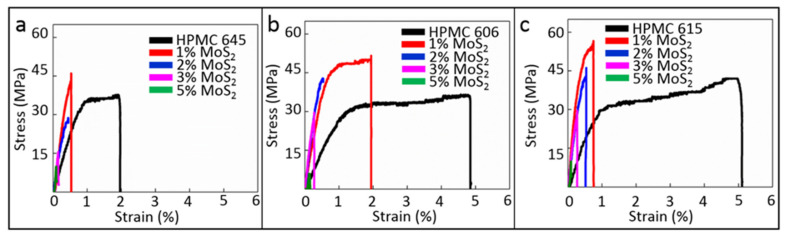
Nano-tensile tester results of the (**a**) MoS_2_/HPMC 645, (**b**) MoS_2_/HPMC 606, and (**c**) MoS_2_/HPMC 615 composites.

The tensile strength, elastic modulus, and percentage elongation of the films were measured to evaluate the improvements in the mechanical properties of composite films compared to those of pure HPMC. The stress–strain curves of the composite films with three different molecular masses (molecular chain lengths) are shown in Figure 2. HPMC with longer molecular chains exhibited better mechanical properties. The addition of small quantities (1%) of MoS_2_ additives enhanced the ultimate tensile strength (UTS) of the film but reduced its ductility. Furthermore, as the amount of MoS_2_ increased, UTS and ductility of the film decreased. This may be because an appropriate amount of additive could form better local bonding with the HPMC matrix. This hypothesis can be verified with the experimental results of FTIR.

**Figure 3:**
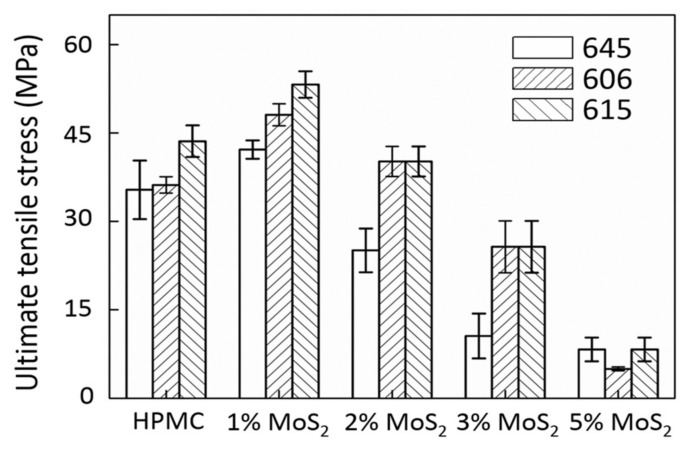
UTS results of MoS_2_/HPMC composites.

**Figure 4:**
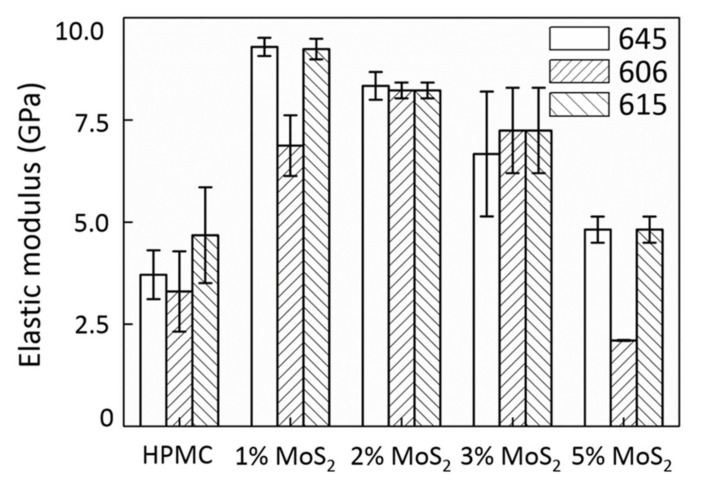
Elastic modulus of MoS_2_/HPMC composites.

**Figure 5:**
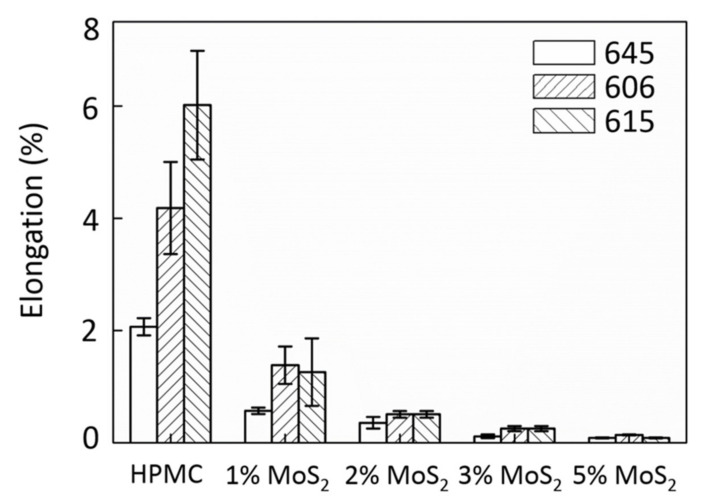
Elongation of MoS_2_/HPMC composites.

Figure 3 shows that the addition of 1 wt% of MoS_2_ provided the best result in terms of UTS, that is, an increase of up to 20%. A further increase in MoS_2_ additives led to a significant decrease in UTS. As shown in Figure 4, the elastic modulus of the MoS_2_/HPMC composite considerably improved when the amount of additive was less than 3%, suggesting that the elastic behavior of the films was strongly affected by a small amount of additives. The elastic moduli of the films containing 1–3% MoS_2_ were dramatically more significant than those of the pure HPMC matrix from 1500 to 30%. Therefore, the addition of MoS_2_ within this range improved the matrix bonding. Based on the elongation (EL) analysis shown in Figure 5, the addition of MoS_2_ embrittled HPMC films and changed these films from ductile to brittle materials. Shi et al. [27] found that the addition of 5–10% MoS_2_ resulted in optimal tribological properties. This is due to the fact that the primary mechanical property in this study is tensile strength, whereas wear and lubrication occur under compressive conditions. As the addition of MoS_2_ particles embrittled an HPMC film, the material broke down when subjected to stress, allowing the MoS_2_ particles to directly contact the wear component. Hence, the attainment of optimal wear resistance and friction coefficients at higher additive ratios (5–10%) was caused by the combination of material breakage with various accommodation mode tribological mechanisms of MoS_2_ [12,34] and the ease with which MoS_2_ synergizes with HPMC to form the transfer layers. However, the breakdown of the material also accelerated film depletion. This is consistent with previous findings, which have reported that the addition of MoS_2_ cannot extend the service life of a film [17].

### 3.3. ATR–FTIR Spectroscopy

**Figure 6:**
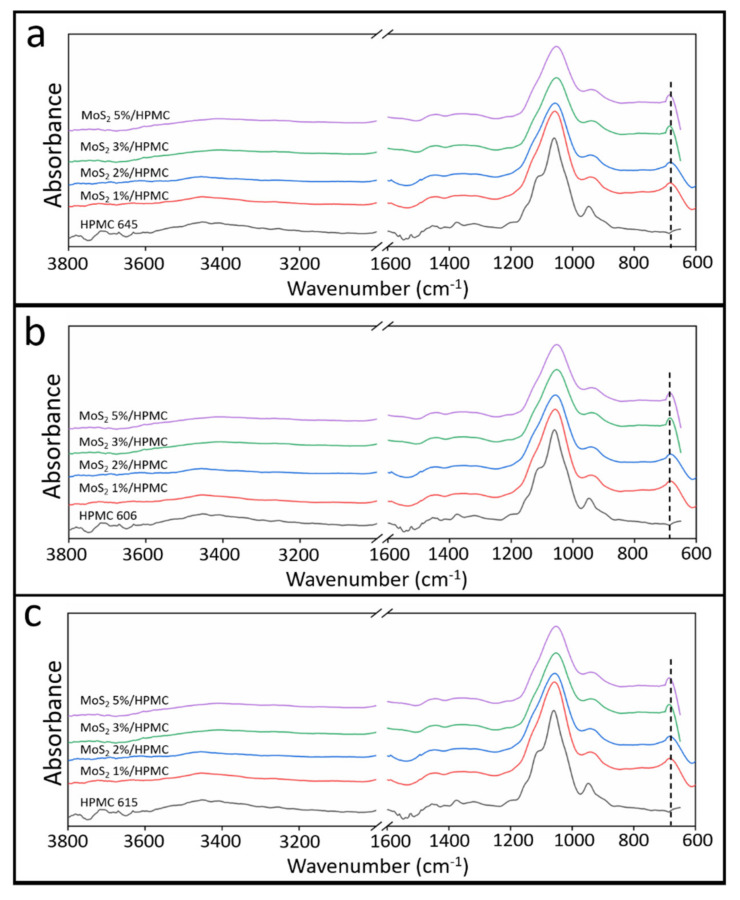
Fourier transform infrared (FTIR) spectra of hydroxypropyl methylcellulose (HPMC): (**a**) 645 composites; (**b**) 606 composites; and (**c**) 615 composites.

The properties of composite materials greatly affect the intermolecular interactions between various components [35,36]. The modulation properties can be enhanced by adjusting intra- and intermolecular interactions between the components [37]. The conventional way of observing the intermolecular interactions involves the use of vibrational spectroscopy [36,38]. However, FTIR spectroscopy provides a quick and straightforward analysis to observe the interaction [39]. Surface functional groups of the composite films were examined to evaluate whether the MoS_2_ particles modulate bonding between the additive and matrix before and after particle addition. Angles of incidence greater than the critical angle result in continuous total reflectance of an infrared beam between the sample surface and the ATR crystal. This internal reflectance creates an evanescent wave that the sample may absorb. The infrared beam spectrum, in which the energy is attenuated after multiple reflections, may then be used to analyze the molecular vibrations on the sample surface. The FTIR spectra of the HPMC and MoS_2_/HPMC composite films are shown in Figure 6. With the appropriate quantity of MoS_2_ additive, minor changes were observed in the intermolecular H bonding and OH bonding (3600–3200 cm^−1^) of pure HPMC, which indicated that the addition of MoS_2_ particles did not reduce the number of molecular bonds. A new band was observed at 690 cm^−1^ (indicated by a dashed line) after the addition of MoS_2_ to the matrix owing to the interaction of S^2−^ with H^+^ in HPMC. MoS_2_ bonded with HPMC and formed molybdenum oxides. The peak at 690 cm^−1^ indicates the formation of MoO_2_ (MoS_2_ oxidized by water) bonding between HPMC and MoS_2_. Based on the change in molecular bonding, an appropriate addition of MoS_2_ particles (1 wt%) increases the bonding between additive particles and the HPMC matrix, thus enhancing the mechanical properties of the composite films. The MoS_2_ additives used in this study have large particle sizes (at the μm level), and longer-chain HPMC (HPMC 615) may establish more bonding with additives, resulting in higher tensile strength, as demonstrated in Figure 2, Figure 3, Figure 4 and Figure 5.

### 3.4. WVP Analysis

**Figure 7:**
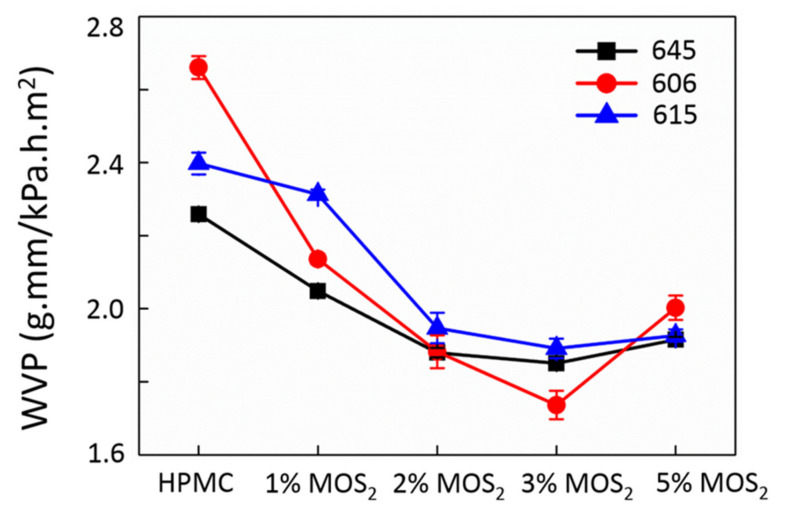
WVP of MoS_2_/HPMC composites.

The significant index (lower values are preferred) for industrial food packaging is the WVP index [8,40]. As MoS_2_ is hydrophobic [11], we predicted that the WVP of the composite films would be reduced. The WVP values of pure HPMC and composite films are shown in Figure 7. The WVP of HPMC films decreased with additives and eventually stabilized beyond a certain level of addition. This demonstrates that the addition of MoS_2_ particles effectively reduces WVP. However, without the addition of a dispersant, WVP eventually reached a limit (approximately 1.9 ± 10% g·mm/kPa·h·m^2^ in this study). A small amount of MoS_2_ additives can succeed in blocking water vapor from entering the composite material due to the intermolecular bonding between the additive and matrix. It is also helpful to reduce the frictional resistance caused by water vapor.

### 3.5. Tribological Behavior of MoS_2_/HPMC Composites

**Figure 8:**
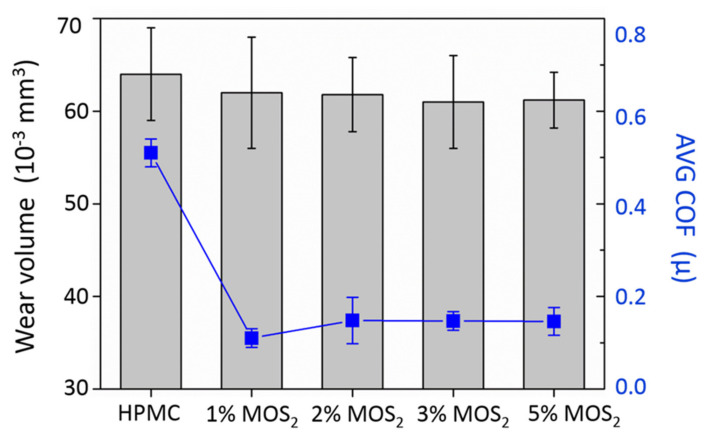
Wear volume and average coefficient of friction of MoS_2_/HPMC composites.

The MoS_2_/HPMC 615 composite film offered the best mechanical properties and was used for subsequent wear tests. As shown in Figure 8, the MoS_2_ additives effectively reduced the friction coefficient by 70%. However, the wear volume hardly reduced. This is in agreement with the study conducted by Shi [17]. The MoS_2_ additive can facilitate the formation of a transfer layer, thereby reducing the coefficient of friction. MoS_2_ with a layered structure caused delamination during abrasion and detached itself from the bonded matrix, resulting in wear. A small amount of wear reduction occurred when MoS_2_ was bonded to the matrix, which led to better load resistance. The deformation of the film surface reduced, and the wear contact area and abrasion volume were lessened. However, the effect of MoS_2_ additives in reducing the wear of the composite film was not apparent.

### 3.6. Third-Body Tribological Mechanism

A previous study had reported that the addition of MoS_2_ results in a transfer layer during abrasion, which can effectively reduce the coefficient of friction [29]. However, the generation of the transfer layer did not fully respond to the slight wear reduction and low coefficient of friction with the MoS_2_ additive. The third-body theory can explain this behavior adequately. With the addition of 1 wt% MoS_2_ additives, UTS increased by 20% and the elastic modulus increased by 1500%, indicating that MoS_2_/HPMC composites are very strong and rigid. When the composite material was subjected to a force, its deformation resistance increased and it provided a better S5M1 (S5 represents the MoS_2_/HPMC composite film; M1 represents the elastic deformation) third-body velocity accommodation mechanism, and a better coefficient of friction was obtained. The main reason for transfer layer formation is the delamination of MoS_2_ [17]; the presence of MoS_2_ in the composite can provide transfer layer formation. In addition, this behavior explains why the coefficient of friction was practically constant with the addition of MoS_2_ in composites. The additives simultaneously increased the load resistance of the composite material and the embrittlement of the coating. Embrittlement coating was more likely to abrade out of the system by a third-body flow during abrasion, resulting in wear [29]. These two mechanisms culminated in a slight wear reduction. 

## 4. Conclusions

MoS_2_/HPMC composite films were successfully prepared using the solvent evaporation method. The mechanical properties, such as the UTS and Young’s modulus, were enhanced dramatically with 1 wt% additive. The addition of MoS_2_ to HPMC strengthened the mechanical and vapor barrier behavior of the HPMC composite because of high interfacial bonding between MoS_2_ and the HPMC matrix. A matrix with a large molecular weight provided more sites for bonding with additives, thus improving the mechanical properties and load resistance. However, embrittlement of the composite film promoted the separation of MoS_2_/HPMC composite material from the film surface, which formed an effective transfer/protection layer and resulted in excellent tribological properties. The properties of HPMC were enhanced when the additives were incorporated. Although FTIR spectra illustrate that intermolecular forces transpire between additive and matrix materials, results from other analytical instruments is essential. The principal components analysis-assisted ATR–FTIR and thermogravimetric analysis can provide additional evidence to make the findings more significant. The application of environmentally friendly materials with improved mechanical properties would be beneficial for the food packaging industry.

## Figures and Tables

**Table 1:** Specifications of hydroxypropyl methylcellulose (HPMC) powders.

Grade	Molecular Weight (g/mol)	Viscosity (mPas)
HPMC 645	20,000	4.5
HPMC 606	35,600	6
HPMC 615	60,000	15

**Table 2:** Material properties of HPMC and MoS_2_.

Material	Degradability	Biofriendly	Light Absorption	Moisture Barrier	Strength	Tribology Properties
HPMC	Good [22]	Good [23]	Bad	Bad	Bad	Bad
MoS_2_	Bad	Good [16]	Good [24]	Good [25]	Good [26]	Good [27]

**Table 3:** Masses of nanoparticle additives used in film and corresponding percent by weight concentration.

**MoS_2_ (g)**	0	1.03	2.06	3.09	5.15
**MoS_2_ (wt%)**	0	1	2	3	5
